# Exploring the impact of physiotherapy on health outcomes in older adults with chronic diseases: a cross-sectional analysis

**DOI:** 10.3389/fpubh.2024.1415882

**Published:** 2024-09-09

**Authors:** Ravi Shankar Reddy, Khalid A. Alahmari, Mastour Saeed Alshahrani, Batool Abdulelah Alkhamis, Jaya Shanker Tedla, Mohammad A. ALMohiza, Basant Hamdy Elrefaey, Ghada M. Koura, Kumar Gular, Hani Hassan Alnakhli, Debjani Mukherjee, Vikram Sreenivasa Rao, Khalid Awad Al-Qahtani

**Affiliations:** ^1^Program of Physical Therapy, Department of Medical Rehabilitation Sciences, College of Applied Medical Sciences, King Khalid University, Abha, Saudi Arabia; ^2^Department of Health Rehabilitation Sciences, College of Applied Medical Sciences, King Saud University, Riyadh, Saudi Arabia; ^3^Department of Anatomy, College of Medicine, King Khalid University, Abha, Saudi Arabia

**Keywords:** physiotherapy, chronic diseases, older adult health, pain management, mobility, functional independence, geriatric care

## Abstract

**Objective:**

This study evaluates the impact of physiotherapy interventions on health outcomes and explores the correlation between physiotherapy session characteristics and improvements in health among older individuals.

**Methods:**

In a cross-sectional design, 384 older adults with chronic conditions such as arthritis, osteoporosis, Chronic Obstructive Pulmonary Disease (COPD), diabetes, and hypertension were recruited.

**Results:**

The proportion of arthritis (39.1%) and hypertension (45.8%) was notably high. Participants receiving physiotherapy showed significant improvements in pain levels (mean reduction from 5.09 to 2.95), mobility scores (improvement from 3.0 to 3.96), and functional independence. A positive correlation was identified between the frequency of physiotherapy sessions and pain reduction (*r* = 0.26, *p* = 0.035), and a stronger correlation between session duration and both pain reduction (*r* = 0.38, *p* = 0.002) and mobility improvement (*r* = 0.43, *p* = 0.001). High satisfaction rates with physiotherapy were reported, and age was found to be a significant negative predictor of health outcomes (Coef. = −0.3402, *p* = 0.0009).

**Conclusion:**

Physiotherapy interventions significantly improve health outcomes in older adults with chronic diseases.

## Introduction

As the global population steadily ages, chronic diseases increasingly dominate the landscape of public health concerns (1). Among the older adult, chronic conditions such as arthritis, osteoporosis, chronic obstructive pulmonary disease (COPD), diabetes, and hypertension not only diminish life quality but also impose substantial burdens on healthcare systems worldwide (2). The World Health Organization estimates that chronic diseases are the leading cause of mortality globally, accounting for 60% of all deaths (3).

Older adult individuals often face multiple chronic conditions simultaneously, complicating treatment and management strategies (4). Arthritis and osteoporosis commonly exhibit concurrent occurrence, particularly among postmenopausal women, thereby affecting mobility and autonomy (5). Similarly, COPD and hypertension often coexist, exacerbating each other and leading to a declining quality of life (6). Understanding the distribution and proportion of these conditions is crucial for effective healthcare planning and intervention (6). Numerous studies have explored the prevalence of chronic diseases in the older adults (6). However, many of these studies are limited by their scope, focusing on single conditions or specific geographical areas (6). There is a distinct need for updated, comprehensive data that encompasses a wider range of chronic conditions and demographic variables (7). Furthermore, while the beneficial effects of physiotherapy on certain chronic conditions have been documented, there is a lack of holistic research that assesses its impact across a variety of health outcomes (7).

Physiotherapy has emerged as a vital component in the management of chronic diseases (8). It plays a crucial role in pain reduction, enhancing mobility, and improving functional independence, which is critical for the older adults to maintain a satisfactory quality of life (8). The American Physical Therapy Association highlights physiotherapy as a safe and effective alternative to medication for managing pain and improving physical function (9). Different physiotherapy techniques, such as strength training, balance exercises, flexibility exercises, and aerobic conditioning, have been employed to address various aspects of chronic diseases (9). Strength training is imperative in the management of osteoporosis, whereas balance exercises play a pivotal role in fall prevention among the older adults (10). However, the effectiveness of these interventions can vary based on their frequency and duration, which has not been thoroughly investigated in previous studies (11).

The nexus between the frequency and duration of physiotherapy sessions and resultant health outcomes in the older adults is an area that warrants more extensive exploration (11). Current literature offers limited guidance on the optimal frequency and duration of physiotherapy sessions to maximize health benefits (12). This knowledge gap is particularly significant given the diverse health challenges faced by the older adults, where physiotherapy plays a crucial role in managing chronic conditions, alleviating pain, and improving overall functional capabilities (13). The frequency of physiotherapy—how often sessions occur—could be a key determinant in the efficacy of treatment (13). There is a need to ascertain whether more frequent sessions lead to more rapid or greater improvements in health outcomes, or if there is a threshold beyond which additional sessions do not yield proportional benefits (13). Similarly, the duration of each session is a critical factor. It remains unclear whether longer sessions provide compounded therapeutic advantages or if shorter, more frequent sessions could offer similar or greater benefits. A nuanced understanding of how these variables interact and influence patient outcomes is essential. It has potential implications for tailoring physiotherapy regimens to individual needs, maximizing therapeutic efficacy (13). This becomes increasingly important in geriatric care, where patients often present with multiple comorbidities and varying degrees of physical capability (14). Developing effective and efficient physiotherapy protocols, based on a thorough understanding of these dynamics, is pivotal in enhancing the quality of life and health outcomes for older adults (14).

This research endeavors to bridge the existing knowledge gaps by conducting an extensive cross-sectional analysis focused on the older adult demographic afflicted with chronic diseases. The study is anchored on two principal objectives: Firstly, to evaluate the efficacy of diverse physiotherapy interventions in influencing key health outcomes, such as pain reduction, mobility enhancement, and functional independence, among older individuals grappling with chronic diseases. This evaluation will involve a detailed examination of how various physiotherapy techniques impact these critical health parameters. Secondly, the research aims to elucidate the relationship between the characteristics of physiotherapy sessions—specifically, their frequency and duration—and the observed improvements in health outcomes, thereby identifying significant predictors that augment the effectiveness of physiotherapy in the management of chronic conditions in the aging population.

## Methods

### Study design

This cross-sectional observational study aimed to assess the impact of physiotherapy interventions on health outcomes in older adults with chronic diseases. Ethical clearance was obtained from the King Khalid University Ethics Committee (protocol number REC# 125–2022), following the guidelines of the Declaration of Helsinki for research involving human participants.

### Study population characteristics

The study focused on individuals aged 65 years and older, who were diagnosed with one or more chronic diseases such as arthritis, osteoporosis, Chronic Obstructive Pulmonary Disease (COPD), diabetes, and hypertension. The proportions of each chronic disease among the 384 participants were as follows: arthritis (39.1%), osteoporosis (29.2%), COPD (20.3%), diabetes (24.0%), and hypertension (45.8%).

### Recruitment process

Participants were recruited from community centers, senior citizen clubs, outpatient clinics, and healthcare facilities. Information sessions were held at these centers to inform potential participants about the study’s aims, the importance of their contribution, and the procedures involved.

### Sampling strategy

A stratified random sampling technique was employed, categorizing the population into different strata based on age, gender, and type of chronic disease. The older population was divided into age groups (e.g., 65–74, 75–84, 85+), and equal representation of male and female participants was aimed for.

### Inclusion and exclusion criteria

Inclusion criteria comprised age 65 years or older, a confirmed diagnosis of one or more of the specified chronic diseases, and the ability to provide informed consent. Exclusion criteria included individuals with acute conditions or recent surgeries that could interfere with physiotherapy interventions, as well as those with cognitive impairments.

### Data collection procedures

Data were collected through structured interviews and questionnaires. These instruments gathered detailed information on demographic characteristics, health status, and physiotherapy interventions received. The questionnaires included validated scales for measuring pain levels, functional mobility, and overall quality of life. To validate the self-reported data on physiotherapy interventions and manage potential bias and recall issues, several strategies were implemented. First, participants were provided with detailed instructions and examples to ensure they understood the questions accurately. The questionnaires used were derived from previously validated instruments in similar studies, enhancing their reliability. To further ensure accuracy, participants were asked to corroborate their responses with personal records, such as appointment logs or treatment diaries, whenever available. Additionally, to minimize recall bias, the data collection focused on the past year, a timeframe deemed reasonable for accurate recall by older individuals. The interviewers were trained to assist participants in recalling specific details by using memory aids and probing questions. To manage potential bias, the study ensured anonymity and confidentiality, encouraging honest and accurate reporting by the participants.

### Questionnaire design and implementation

The study employed a detailed questionnaire titled “Questionnaire for Assessing the Impact of Physiotherapy Interventions in Older Adults with Chronic Diseases” to collect demographic data and health status assessments, including physical and mental health, mobility, pain levels, and fall frequency. Participants also provided information on the types and frequencies of physiotherapy interventions they received, along with their satisfaction levels. Changes in mobility, pain levels, functional independence, and quality of life resulting from physiotherapy were assessed using Likert scale questions. Additionally, an open-ended section allowed participants to share additional comments and experiences related to physiotherapy and its effects on their health.

### Questionnaire validation and administration

The questionnaire validation process involved a pilot study with older adults diagnosed with chronic diseases to ensure its comprehensiveness, clarity, and relevance. Feedback from participants led to modifications for improved clarity and precision. Following refinement, the questionnaire was administered using both face-to-face and electronic methods to accommodate participants’ preferences and accessibility needs, ensuring inclusivity and ease of access.

### Physiotherapy intervention assessment

Participants were extensively questioned about their participation in physiotherapy sessions over the past year, detailing the frequency, duration, and types of interventions received. The inquiry covered various physiotherapy modalities such as strength training, balance exercises, flexibility exercises, aerobic conditioning, and pain management techniques, aiming for a comprehensive assessment of participants’ physiotherapy engagement and experiences.

### Data analysis

The data analysis for our cross-sectional study, aimed at evaluating the impact of community-based physiotherapy interventions on chronic disease management in the older adults, was conducted using SPSS version 24 with statistical significance set at *p* < 0.05. Missing data were minimal and were addressed using multiple imputation techniques, specifically predictive mean matching. Outliers were identified through visual inspections (box plots, scatter plots) and statistical methods (Z-scores). Data points with Z-scores exceeding ±3 was further examined for errors, and genuine outliers were retained. Outliers were identified visually and statistically to ensure data robustness. Normality assessments confirmed parametric test validity. Detailed demographic and baseline health status descriptions were provided. Independent samples t-tests were used to compare health outcomes, such as pain levels, mobility scores, and functional independence, between participants who received physiotherapy interventions and those who did not. One-way ANOVA tests compared health outcomes across different types of physiotherapy interventions. Post-hoc tests were conducted following ANOVA to identify specific group differences. Pearson’s correlation coefficients were calculated to explore relationships between session frequency/duration and health outcomes. Multiple linear regression analyses identified significant predictors while adjusting for potential confounders.

## Results

Table 1 provides a demographic overview and baseline characteristics of 384 participants.

**Table 1:** Demographic and baseline characteristics of participants (*N* = 384).

Variable	Total (*n* = 384)	Men (*n* = 192)	Women (*n* = 192)
	*n* (%)	*n* (%)	*n* (%)
**Age (years)**			
Mean (SD)	72.5 (6.3)	72.8 (6.1)	72.2 (6.5)
Range	65–90	65–90	65–89
**Living situation**			
Alone	154 (40.1%)	75 (39.1%)	79 (41.1%)
With family	176 (45.8%)	89 (46.4%)	87 (45.3%)
In a care facility	54 (14.1%)	28 (14.6%)	26 (13.5%)
**Marital status**			
Married	212 (55.2%)	108 (56.3%)	104 (54.2%)
Widowed	110 (28.6%)	40 (20.8%)	70 (36.5%)
Single/never married	32 (8.3%)	22 (11.5%)	10 (5.2%)
Divorced/separated	30 (7.8%)	22 (11.5%)	8 (4.2%)
**Education level**			
No formal education	48 (12.5%)	22 (11.5%)	26 (13.5%)
Primary school	110 (28.6%)	50 (26.0%)	60 (31.3%)
Secondary school	142 (37.0%)	74 (38.5%)	68 (35.4%)
Higher education	84 (21.9%)	46 (24.0%)	38 (19.8%)
**Primary chronic disease**			
Arthritis	150 (39.1%)	68 (35.4%)	82 (42.7%)
Osteoporosis	112 (29.2%)	42 (21.9%)	70 (36.5%)
COPD	78 (20.3%)	45 (23.4%)	33 (17.2%)
Diabetes	92 (24.0%)	49 (25.5%)	43 (22.4%)
Hypertension	176 (45.8%)	90 (46.9%)	86 (44.8%)
BMI (kg/m^2^) mean (SD)	27.8 (4.2)	27.6 (4.1)	28.0 (4.3)
Functional mobility (score 1–5) mean (SD)	3.2 (1.1)	3.3 (1.0)	3.1 (1.2)
Pain level (score 0–10) mean (SD)	4.5 (2.3)	4.3 (2.2)	4.7 (2.4)
**Falls in the past year**			
0 falls	276 (71.9%)	138 (71.9%)	138 (71.9%)
1–2 falls	86 (22.4%)	42 (21.9%)	44 (22.9%)
3+ falls	22 (5.7%)	12 (6.3%)	10 (5.2%)
**Physiotherapy in past year**			
Yes	264 (68.8%)	130 (67.7%)	134 (69.8%)
No	120 (31.2%)	62 (32.3%)	58 (30.2%)
**Smoking status**			
Current smoker	48 (12.5%)	30 (15.6%)	18 (9.4%)
Former smoker	116 (30.2%)	60 (31.3%)	56 (29.2%)
Never smoked	220 (57.3%)	102 (53.1%)	118 (61.5%)
**Exercise frequency (per week)**			
None	134 (34.9%)	60 (31.3%)	74 (38.5%)
1–2 times	116 (30.2%)	58 (30.2%)	58 (30.2%)
3–4 times	86 (22.4%)	48 (25.0%)	38 (19.8%)
5 or more times	48 (12.5%)	26 (13.5%)	22 (11.5%)
Mental health status (score 1–5) mean (SD)	3.8 (1.2)	3.7 (1.3)	3.9 (1.1)
Chronic pain duration (years) mean (SD)	5.4 (3.2)	5.2 (3.0)	5.6 (3.4)
Physiotherapy satisfaction (score 1–5) mean (SD)	4.1 (1.0)	4.0 (1.1)	4.2 (0.9)

SD, standard deviation; COPD, chronic obstructive pulmonary disease; BMI, body mass index.

The study included 384 older participants diagnosed with one or more chronic diseases. The proportions of participants with each chronic disease were as follows: arthritis (39.1%), osteoporosis (29.2%), Chronic Obstructive Pulmonary Disease (COPD) (20.3%), diabetes (24.0%), and hypertension (45.8%). These proportions reflect the distribution of chronic diseases within our selected cohort and do not indicate the true proportion of these conditions in the general population.

Table 2 shows the types and frequency of physiotherapy interventions among participants. Strength training was the most prevalent, with 96.1% of participants receiving it at varying frequencies, including daily (13.6%), weekly (33.0%), and as needed (33.3%). Balance and coordination exercises were performed by 68.0% of participants, with 22.0% doing it weekly and 33.0% as needed. Flexibility exercises were administered to 72.1% of participants, primarily weekly (33.3%) and monthly (30.7%). Aerobic conditioning was used by 67.2% of participants, with 29.2% practicing it daily and 30.3% as needed. Pain management techniques were employed by 88.8% of participants, with 29.9% using them weekly and 31.1% as needed. Other interventions were reported by 73.9% of participants, with 37.5% receiving them daily and 33.3% weekly.

**Table 2:** Types and frequency of physiotherapy interventions.

Type of physiotherapy intervention	Daily (*n* (%))	Weekly (*n* (%))	Monthly (*n* (%))	Less than monthly (*n* (%))	As needed (*n* (%))	Total number of participants receiving each mode (*n* (%))
Strength training	36 (13.6%)	87 (33.0%)	70 (26.5%)	88 (33.3%)	88 (33.3%)	369 (96.1%)
Balance and coordination exercises	12 (4.5%)	58 (22.0%)	65 (24.8%)	39 (14.8%)	87 (33.0%)	261 (68.0%)
Flexibility exercises	46 (17.4%)	88 (33.3%)	81 (30.7%)	37 (14.0%)	25 (9.5%)	277 (72.1%)
Aerobic conditioning	77 (29.2%)	72 (27.3%)	9 (3.4%)	20 (7.6%)	80 (30.3%)	258 (67.2%)
Pain management techniques	69 (26.1%)	79 (29.9%)	47 (17.8%)	64 (24.2%)	82 (31.1%)	341 (88.8%)
Other	99 (37.5%)	88 (33.3%)	49 (18.6%)	29 (11.0%)	19 (7.2%)	284 (73.9%)

This study compares health outcomes between participants who received physiotherapy interventions and those who did not. Results indicate significant differences in pain levels, mobility, and functional independence. Participants who underwent physiotherapy had lower mean pain levels and higher mobility and functional independence scores compared to those who did not. The physiotherapy group showed effective pain management, improved mobility, and enhanced autonomy in daily activities. Standard deviations were similar between groups, indicating consistent variability in responses.

**Figure 1:**
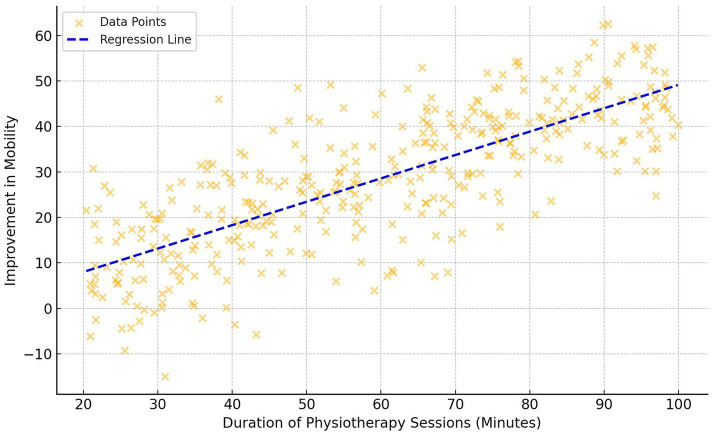
Scatter plot—duration of physiotherapy sessions vs. improvement in mobility.

**Figure 2:**
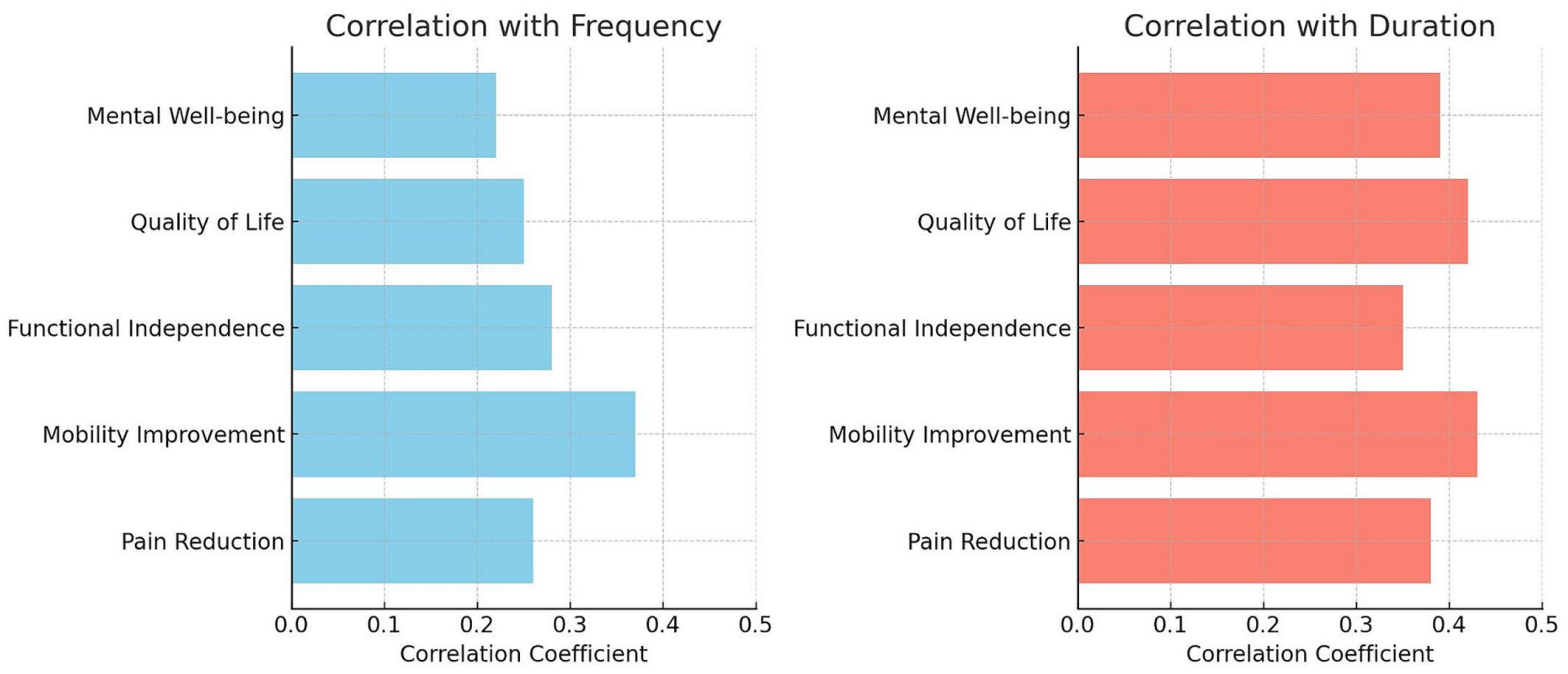
Correlations between physiotherapy frequency/duration and various health outcomes in older adults.

Figure 1 illustrates the relationship between physiotherapy session length and mobility improvement in 384 participants. The scatter plot displays a positive correlation, indicating that longer sessions are linked to greater mobility enhancements. The spread of data points around the regression line highlights individual variability in response to session duration. Table 3 and Figure 2 demonstrate significant correlations between physiotherapy session frequency/duration and health outcomes. More frequent sessions correlate moderately with pain reduction (*r* = 0.26, *p* = 0.035), while longer sessions show a stronger correlation (*r* = 0.38, *p* = 0.002). Mobility improvement correlates with both frequency (*r* = 0.37, *p* = 0.020) and duration (*r* = 0.43, *p* = 0.001). Functional independence positively correlates with session frequency (*r* = 0.28, *p* = 0.050) and duration (*r* = 0.35, *p* = 0.003). Quality of life correlates with frequency (*r* = 0.25, *p* = 0.030) and duration (*r* = 0.42, *p* = 0.005), while mental well-being shows correlations of *r* = 0.22 (*p* = 0.040) for frequency and *r* = 0.39 (*p* = 0.015) for duration.

**Table 3:** Correlation between frequency/duration of physiotherapy and health outcomes.

Outcome	Correlation with frequency	*p*-value frequency	Correlation with duration	*p*-value duration
Pain reduction	0.26	0.035	0.38	0.002
Mobility improvement	0.37	0.020	0.43	0.001
Functional independence	0.28	0.050	0.35	0.003
Quality of life	0.25	0.030	0.42	0.005
Mental well-being	0.22	0.040	0.39	0.015

Among 384 study participants, 33.6% reported being “Very Satisfied” with physiotherapy, while 38% were “Satisfied.” Additionally, 16.4% remained “Neutral,” 7.3% felt “Dissatisfied,” and 4.7% were “Very Dissatisfied.” These findings highlight varying perceptions of physiotherapy effectiveness among older individuals with chronic diseases.

**Figure 3:**
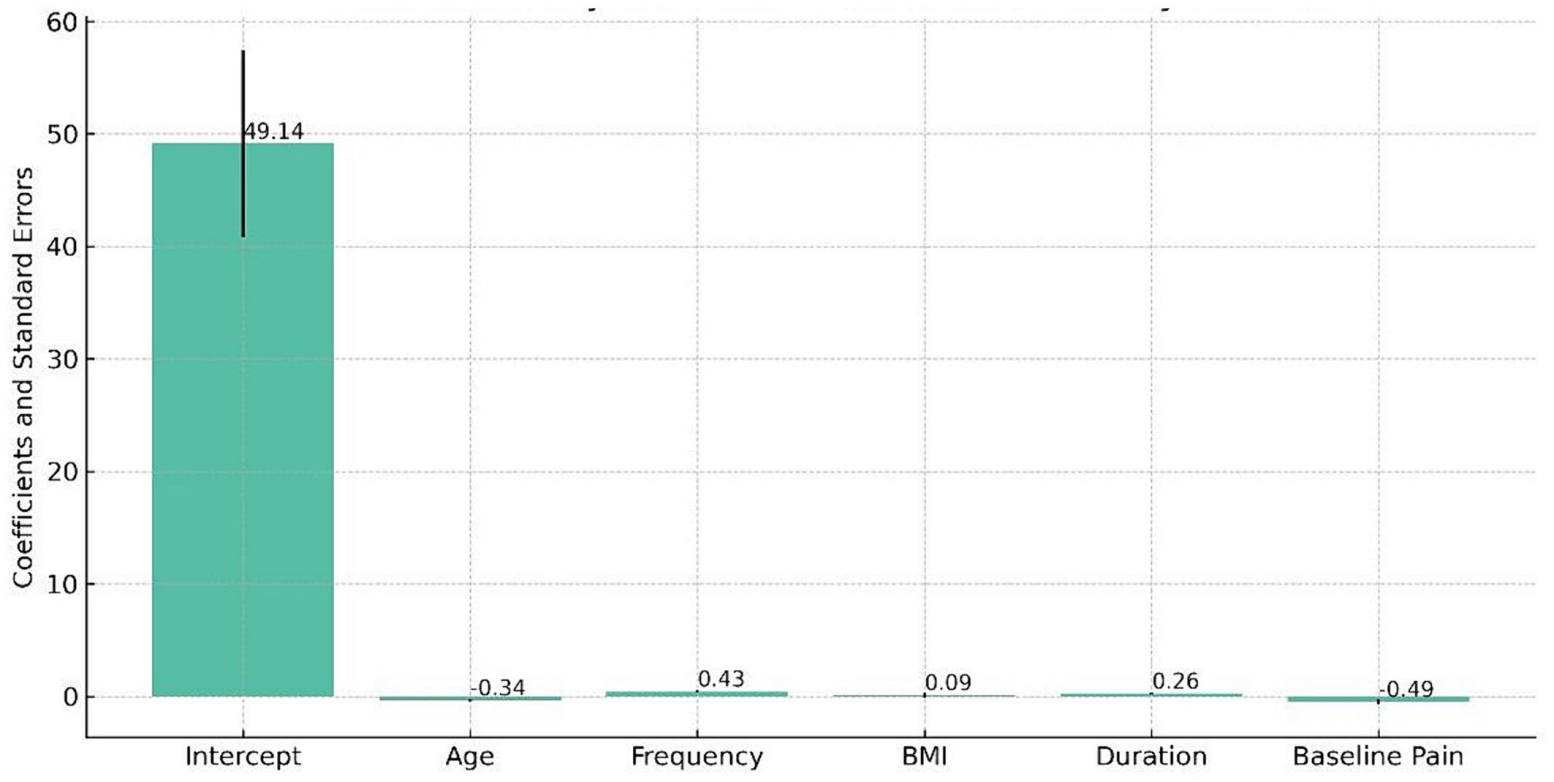
Multivariate regression analysis of predictors affecting health outcomes in older adults with chronic diseases.

The regression analysis in this study revealed important insights into health outcomes (Figure 3). The baseline health outcome, with all predictors at zero, was high (Coef. = 49.1367, *p* < 0.001). Age negatively impacted health outcomes, with each additional year of age associated with a decrease in the health outcome score by 0.340 units (*p* < 0.001). This indicates that as participants age, their scores on health outcome measures, such as pain levels, mobility, and functional independence, tend to decrease. More frequent physiotherapy sessions positively influenced health outcomes, each additional session per week improving the score by 0.4261 points (*p* = 0.0009). BMI, however, was not a significant predictor (Coef. = 0.0947, *p* = 0.7127). Longer physiotherapy session durations also positively affected health outcomes, with each extra minute increasing the score by 0.2555 points (*p* < 0.0001). Baseline pain level showed a trend toward a negative impact on health outcomes (Coef. = −0.4896, *p* = 0.0579).

## Discussion

The findings of this study regarding the high proportion of arthritis and hypertension among older adults corroborate with the existing body of research, notably the study by Wong et al. (15), which identified these conditions as notably common in the aging population (15). This proportion is reflective of the age-related degenerative processes and lifestyle factors that typically contribute to the development and progression of these chronic diseases (16). Arthritis, as characterized by joint pain and stiffness, tends to escalate with age due to the wear and tear of joints over time (17). Similarly, hypertension, often a result of long-term lifestyle choices and genetic predispositions, becomes more common as the body’s ability to regulate blood pressure diminishes with age (17).

The demographic diversity observed in our study, encompassing a balanced gender distribution and a wide age range, provided a comprehensive view of chronic disease distribution. This aspect is crucial, as demographic variables can significantly influence both the proportion and the management of chronic diseases (18). For instance, He et al. (19) highlighted that certain chronic conditions might be more prevalent in one gender over the other, or certain age groups might be more susceptible to specific diseases (19). The variation in disease manifestation and response to treatment across different demographic groups underscores the importance of personalized healthcare approaches (20). Moreover, our study’s findings contribute to a deeper understanding of the epidemiology of chronic diseases in older adults (20). The demographic diversity observed in our study, encompassing a balanced gender distribution and a wide age range, provided a comprehensive view of chronic disease distribution. Understanding the proportion and impact of chronic diseases in different demographic groups can help in designing targeted interventions. For instance, knowing that certain conditions are more prevalent in specific age groups or genders allows for more precise healthcare planning and resource allocation, ultimately improving the effectiveness of interventions (21). The significant improvements in pain levels, mobility, and functional independence observed in participants undergoing physiotherapy interventions in this study are a testament to the effectiveness of these interventions in older adults (22, 23). These findings are in line with those of Raymond et al. (24), who reported similar benefits of physiotherapy in older adults. Their research emphasized the role of targeted physiotherapy in not only reducing pain but also enhancing mobility, critical factors in maintaining independence and quality of life in older adults (25).

Our study’s findings are also consistent with those of Thompson et al. (26), who highlighted the personalized nature of effective physiotherapy (26). The variability in intervention types, such as strength training, balance exercises, and flexibility routines, underscores the necessity of personalized treatment plans (27). This personalization caters to the unique needs of each patient, considering factors such as the specific chronic condition(s) present, the patient’s baseline physical capabilities, and their progress over time (27). Moreover, the impact of physiotherapy on functional independence is particularly noteworthy (28). Functional independence is vital for older adults, as it directly affects their ability to perform daily activities and maintain a sense of autonomy (28). The improvement in this area, as demonstrated in our study, suggests that physiotherapy can play a crucial role in prolonging and enhancing the independent living of older individuals (29).

The positive correlation found in this study between the frequency and duration of physiotherapy sessions and improved health outcomes, particularly in pain reduction and mobility, provides valuable insights into the dynamics of physiotherapy efficacy. This correlation is in line with the research conducted by Brown et al. (30), who emphasized the crucial role that both the regularity and the length of therapy sessions play in maximizing therapeutic benefits. Their findings highlighted that consistent and prolonged engagement in physiotherapy could lead to more significant improvements in patient health, a conclusion that our study corroborates (31). One plausible explanation for this correlation is the cumulative effect of physiotherapy interventions (32). Regular and extended sessions allow for the progressive building of strength, flexibility, and endurance, which are essential for pain management and mobility enhancement (32). This is particularly true in the context of chronic conditions prevalent in older adult populations, where gradual and consistent intervention can be more effective (33). Longer sessions provide therapists with the time to apply a wider range of techniques and address multiple aspects of the patient’s condition, which can be particularly beneficial for complex chronic diseases (34–36). However, it is important to consider individual variability in response to physiotherapy. While the general trend indicates positive outcomes with increased frequency and duration, individual patient factors such as specific health conditions, overall fitness levels, and personal preferences can influence the optimal physiotherapy regimen (36, 37).

The high satisfaction rates with physiotherapy interventions observed in this study are in harmony with the findings of Badau et al. (38). They too reported a general preference for and positive reception of physiotherapy among the older adults, highlighting the significance of these interventions in this age group (38). This positive perception of physiotherapy could be attributed to the tangible improvements in pain management and mobility that patients experience, which directly impact their daily functioning and quality of life (39). The results emphasize the criticality of patient-centered approaches in managing chronic diseases, especially in the older adults (40). Such approaches, which focus on the preferences and needs of the patient, are crucial for ensuring adherence to treatment plans and for achieving the best possible outcomes (40). Furthermore, the role of age as a predictor of health outcomes, as revealed in our study, provides an important perspective on the challenges faced by the older adults population (41).

The age-related decline in physiological functions, such as reduced muscle strength, decreased bone density, and diminished cardiovascular and respiratory function, contribute to this trend (42, 43). Additionally, the psychological aspects of aging, such as the increased proportion of loneliness and depression, can also have a significant impact on health outcomes (43). It is therefore imperative that healthcare providers and caregivers tailor their approaches to address these age-related changes effectively (44). The connection between age and health outcomes also underscores the importance of early intervention and preventive measures in the management of chronic diseases (45). By addressing risk factors and initiating treatment early, it may be possible to mitigate the impact of these diseases as individuals age (46). This proactive approach can be instrumental in improving long-term health outcomes and maintaining a higher quality of life for the older adults (46).

This study has several limitations that need to be acknowledged. First, the cross-sectional design of the study limits our ability to establish causality between physiotherapy interventions and health outcomes. Longitudinal studies would be necessary to confirm the causal relationships suggested by our findings. Second, the reliance on self-reported data introduces the potential for recall bias. Although we implemented strategies such as using validated questionnaires, memory aids, and corroboration with personal records, recall bias cannot be entirely eliminated. Participants might have misremembered or misreported the frequency and duration of physiotherapy sessions, as well as other health-related information. Third, while we used multiple linear regression models and propensity score matching to control for confounding factors, residual confounding may still be present. Factors such as the severity of the chronic disease, variations in physiotherapy techniques, and adherence to physiotherapy protocols could influence the outcomes but were not fully accounted for in our analyses. Fourth, our inclusion criteria focused on older individuals with specific chronic diseases who were able to provide informed consent. This may have resulted in a selection bias, as those with severe cognitive impairments or other acute conditions were excluded. Consequently, our findings may not be generalizable to all older individuals with chronic diseases. Fifth, the study’s generalizability is further limited by the recruitment of participants from community centers, senior citizen clubs, outpatient clinics, and healthcare facilities. While we aimed to capture a diverse sample, the findings may not be fully representative of the broader population of older individuals with chronic diseases, particularly those in different geographical locations or healthcare settings. Lastly, the observational nature of our study means that it cannot provide a true estimate of the proportion of chronic diseases in the general population. Our sample was not randomly selected from the entire population, but rather from specific community and healthcare settings, which may limit the applicability of our results to the general population.

## Conclusion

This study provides valuable insights into the effectiveness of physiotherapy in managing chronic diseases among the older population. It conclusively demonstrates that regular physiotherapy interventions lead to significant improvements in pain management, mobility, and overall functional independence in older individuals with chronic conditions, emphasizing the importance of integrating physiotherapy into standard care regimens for this demographic. Furthermore, the research highlights the proportion of common chronic diseases like arthritis and hypertension among older adults, while also identifying a positive correlation between the frequency and duration of physiotherapy sessions and health outcomes. Despite its limitations, including a cross-sectional design and reliance on self-reported data, the study underscores the importance of further research to validate these findings and enhance our understanding of the long-term impact of physiotherapy interventions on older adults with chronic diseases.

## Data availability statement

The datasets presented in this study can be found in online repositories. The names of the repository/repositories and accession number(s) can be found below: Zenodo: DOI: 10.5281/zenodo.10781036.

## Ethics statement

The studies involving humans were approved by King Khalid University Ethics committee. The studies were conducted in accordance with the local legislation and institutional requirements. The participants provided their written informed consent to participate in this study.

## Author contributions

RR: Conceptualization, Data curation, Formal analysis, Funding acquisition, Methodology, Supervision, Writing – original draft, Writing – review & editing. KA: Conceptualization, Data curation, Methodology, Writing – original draft, Writing – review & editing. MSA: Conceptualization, Data curation, Methodology, Writing – original draft, Writing – review & editing. BA: Conceptualization, Data curation, Methodology, Writing – original draft, Writing – review & editing. JT: Conceptualization, Data curation, Methodology, Writing – original draft, Writing – review & editing. MAA: Conceptualization, Data curation, Methodology, Writing – original draft, Writing – review & editing. BE: Conceptualization, Data curation, Methodology, Writing – original draft, Writing – review & editing. GK: Conceptualization, Data curation, Methodology, Writing – original draft, Writing – review & editing. KG: Conceptualization, Data curation, Methodology, Writing – original draft, Writing – review & editing. HA: Conceptualization, Data curation, Methodology, Writing – original draft, Writing – review & editing. DM: Data curation, Methodology, Writing – review & editing, Conceptualization, Writing – original draft. VR: Conceptualization, Data curation, Writing – review & editing, Methodology, Writing – original draft. KA-Q: Conceptualization, Methodology, Writing – original draft, Data curation, Writing – review & editing.

## References

[1] Briggs AM Shiffman J Shawar YR Åkesson K Ali N Woolf AD. Global health policy in the 21st century: challenges and opportunities to arrest the global disability burden from musculoskeletal health conditions. Best Pract Res Clin Rheumatol. (2020) 34:101549. doi: 10.1016/j.berh.2020.101549 32713802 PMC7377715

[2] Huang Y-L Tsay W-I Her S-H Ho C-H Tsai K-T Hsu C-C. Chronic pain and use of analgesics in the elderly: a nationwide population-based study. Arch Med Sci. (2020) 16:627–34. doi: 10.5114/aoms.2020.92894, PMID: 32399112 PMC7212229

[3] Girum T Mesfin D Bedewi J Shewangizaw M. The burden of noncommunicable diseases in Ethiopia, 2000–2016: analysis of evidence from global burden of disease study 2016 and global health estimates 2016. Int J Chron Dis. (2020) 2020:1–10. doi: 10.1155/2020/3679528, PMID: 32149073 PMC7053448

[4] Soriano JB Kendrick PJ Paulson KR Gupta V Abrams EM Adedoyin RA. Prevalence and attributable health burden of chronic respiratory diseases, 1990–2017: a systematic analysis for the global burden of Disease study 2017. Lancet Respir Med. (2020) 8:585–96. doi: 10.1016/S2213-2600(20)30105-3, PMID: 32526187 PMC7284317

[5] Laskou F Fuggle NR Patel HP Jameson K Cooper C Dennison E. Associations of osteoporosis and sarcopenia with frailty and multimorbidity among participants of the Hertfordshire cohort study. J Cachexia Sarcopenia Muscle. (2022) 13:220–9. doi: 10.1002/jcsm.12870, PMID: 34873876 PMC8818662

[6] Lázár Z Horváth A Tomisa G Tamási L Müller V. Impact of clinical factors on generic and disease-specific quality of life in COPD and asthma-COPD overlap with exacerbations. Pulmonary Med. (2020) 2020:1–9. doi: 10.1155/2020/6164343, PMID: 32789027 PMC7334771

[7] Aisanov Z Khaltaev N. Management of cardiovascular comorbidities in chronic obstructive pulmonary disease patients. J Thorac Dis. (2020) 12:2791–802. doi: 10.21037/jtd.2020.03.60, PMID: 32642187 PMC7330365

[8] Ehrman JK Gordon PM Visich PS Keteyian SJ. Clinical exercise physiology: exercise management for chronic diseases and special Populations. Champaign, Illinois, USA: Human Kinetics (2022).

[9] Yadav V Naqvi WM Burhani T. Pandemics and physiotherapy: an overview of the role of the physiotherapists in restoring functions and quality of life. Int J Res Pharm Sci. (2020) 11:1898–901. doi: 10.26452/ijrps.v11iSPL1.4550

[10] Pergolizzi JV LeQuang JA. Rehabilitation for low back pain: a narrative review for managing pain and improving function in acute and chronic conditions. Pain Ther. (2020) 9:83–96. doi: 10.1007/s40122-020-00149-5, PMID: 32006236 PMC7203283

[11] Severin R Sabbahi A Arena R Phillips SA. Precision medicine and physical therapy: a healthy living medicine approach for the next century. Phys Ther. (2022) 102:pzab253. doi: 10.1093/ptj/pzab253 34718788

[12] Götte M Gauß G Dirksen U Driever PH Basu O Baumann FT. Multidisciplinary network ActiveOncoKids guidelines for providing movement and exercise in pediatric oncology: consensus-based recommendations. Pediatr Blood Cancer. (2022) 69:e29953. doi: 10.1002/pbc.29953, PMID: 36073842

[13] Daley D Payne LP Galper J Cheung A Deal L Despres M. Clinical guidance to optimize work participation after injury or illness: the role of physical therapists: clinical practice guidelines linked to the international classification of functioning, disability and health from the academy of Orthopaedic physical therapy of the American Physical Therapy Association. J Orthop Sports Phys Ther. (2021) 51:CPG1–CPG102. doi: 10.2519/jospt.2021.0303 34338006

[14] Teo PL Bennell KL Lawford BJ Egerton T Dziedzic KS Hinman RS. Physiotherapists may improve management of knee osteoarthritis through greater psychosocial focus, being proactive with advice, and offering longer-term reviews: a qualitative study. J Physiother. (2020) 66:256–65. doi: 10.1016/j.jphys.2020.09.005, PMID: 33036932

[15] Wong CK Mak RY Kwok TS Tsang JS Leung MY Funabashi M. Prevalence, incidence, and factors associated with non-specific chronic low back pain in community-dwelling older adults aged 60 years and older: a systematic review and meta-analysis. J Pain. (2022) 23:509–34. doi: 10.1016/j.jpain.2021.07.012, PMID: 34450274

[16] Giummarra MJ Tardif H Blanchard M Tonkin A Arnold CA. Hypertension prevalence in patients attending tertiary pain management services, a registry-based Australian cohort study. PLoS One. (2020) 15:e0228173. doi: 10.1371/journal.pone.0228173, PMID: 31978196 PMC6980551

[17] Jiang C-h Zhu F Qin T-t. Relationships between chronic diseases and depression among middle-aged and elderly people in China: a prospective study from CHARLS. Curr. Med Sci. (2020) 40:858–70. doi: 10.1007/s11596-020-2270-5 33123901

[18] Sharpe L Michalowski M Richmond B Menzies R Shaw J. Fear of progression in chronic illnesses other than cancer: a systematic review and meta-analysis of a transdiagnostic construct. Health Psychol Rev. (2023) 17:301–20. doi: 10.1080/17437199.2022.2039744, PMID: 35132937

[19] He L Biddle SJ Lee JT Duolikun N Zhang L Wang Z. The prevalence of multimorbidity and its association with physical activity and sleep duration in middle aged and elderly adults: a longitudinal analysis from China. Int J Behav Nutr Phys Act. (2021) 18:1–12. doi: 10.1186/s12966-021-01150-7 34112206 PMC8194125

[20] Wang W Yan Y Guo Z Hou H Garcia M Tan X. All around suboptimal health—a joint position paper of the suboptimal health study consortium and European Association for Predictive, Preventive and Personalised Medicine. EPMA J. (2021) 12:403–33. doi: 10.1007/s13167-021-00253-2 34539937 PMC8435766

[21] Van der Schaar M Alaa AM Floto A Gimson A Scholtes S Wood A. How artificial intelligence and machine learning can help healthcare systems respond to COVID-19. Mach Learn. (2021) 110:1–14. doi: 10.1007/s10994-020-05928-x, PMID: 33318723 PMC7725494

[22] Lima CA Sherrington C Guaraldo A Moraes SA Varanda RR Melo JA. Effectiveness of a physical exercise intervention program in improving functional mobility in older adults after hip fracture in later stage rehabilitation: protocol of a randomized clinical trial (REATIVE study). BMC Geriatr. (2016) 16:1–6. doi: 10.1186/s12877-016-0370-7 27894271 PMC5126860

[23] Martínez-Velilla N Casas-Herrero A Zambom-Ferraresi F de Asteasu MLS Lucia A Galbete A. Effect of exercise intervention on functional decline in very elderly patients during acute hospitalization: a randomized clinical trial. JAMA Intern Med. (2019) 179:28–36. doi: 10.1001/jamainternmed.2018.4869, PMID: 30419096 PMC6583412

[24] Raymond MJ Jeffs KJ Winter A Soh S-E Hunter P Holland AE. The effects of a high-intensity functional exercise group on clinical outcomes in hospitalised older adults: an assessor-blinded, randomised-controlled trial. Age Ageing. (2017) 46:208–13. doi: 10.1093/ageing/afw215, PMID: 27932360

[25] Lihavainen K Sipilä S Rantanen T Kauppinen M Sulkava R Hartikainen S. Effects of comprehensive geriatric assessment and targeted intervention on mobility in persons aged 75 years and over: a randomized controlled trial. Clin Rehabil. (2012) 26:314–26. doi: 10.1177/0269215511423269, PMID: 22007041

[26] Killingback C Thompson M Chipperfield S Clark C Williams J. Physiotherapists’ views on their role in self-management approaches: a qualitative systematic review. Physiother Theory Pract. (2022) 38:2134–48. doi: 10.1080/09593985.2021.1911011, PMID: 33813990

[27] Koh LH Hagger MS Goh VH Hart WG Gucciardi DF. Effects of a brief action and coping planning intervention on completion of preventive exercises prescribed by a physiotherapist among people with knee pain. J Sci Med Sport. (2017) 20:723–8. doi: 10.1016/j.jsams.2017.02.008, PMID: 28396206

[28] De Vries N Van Ravensberg C Hobbelen J Rikkert MO Staal J Nijhuis-Van der Sanden M. Effects of physical exercise therapy on mobility, physical functioning, physical activity and quality of life in community-dwelling older adults with impaired mobility, physical disability and/or multi-morbidity: a meta-analysis. Ageing Res Rev. (2012) 11:136–49. doi: 10.1016/j.arr.2011.11.002 22101330

[29] Auais MA Eilayyan O Mayo NE. Extended exercise rehabilitation after hip fracture improves patients’ physical function: a systematic review and meta-analysis. Phys Ther. (2012) 92:1437–51. doi: 10.2522/ptj.20110274, PMID: 22822235

[30] Brown J Luderowski A Namusisi-Riley J Moore-Shelley I Bolton M Bolton D. Can a community-led intervention offering social support and health education improve maternal health? A repeated measures evaluation of the pact project run in a socially deprived London borough. Int J Environ Res Public Health. (2020) 17:2795. doi: 10.3390/ijerph17082795, PMID: 32325635 PMC7215628

[31] Miller KE Koppenol-Gonzalez GV Arnous M Tossyeh F Chen A Nahas N. Supporting Syrian families displaced by armed conflict: a pilot randomized controlled trial of the caregiver support intervention. Child Abuse Negl. (2020) 106:104512. doi: 10.1016/j.chiabu.2020.104512, PMID: 32408022

[32] Stevens NR Miller ML Soibatian C Otwell C Rufa AK Meyer DJ. Exposure therapy for PTSD during pregnancy: a feasibility, acceptability, and case series study of narrative exposure therapy (NET). BMC Psychol. (2020) 8:1–18. doi: 10.1186/s40359-020-00503-4 33298159 PMC7727253

[33] Disease WHON Cluster MH. Innovative care for chronic conditions: building blocks for action. Geneva, Switzerland: global report World Health Organization (2002).

[34] Hills R Kitchen S. Satisfaction with outpatient physiotherapy: focus groups to explore the views of patients with acute and chronic musculoskeletal conditions. Physiother Theory Pract. (2007) 23:1–20. doi: 10.1080/09593980601023705, PMID: 17454795

[35] Nijs J Roussel N Van Wilgen CP Köke A Smeets R. Thinking beyond muscles and joints: therapists’ and patients’ attitudes and beliefs regarding chronic musculoskeletal pain are key to applying effective treatment. Man Ther. (2013) 18:96–102. doi: 10.1016/j.math.2012.11.001, PMID: 23273516

[36] Wilson S Chaloner N Osborn M Gauntlett-Gilbert J. Psychologically informed physiotherapy for chronic pain: patient experiences of treatment and therapeutic process. Physiotherapy. (2017) 103:98–105. doi: 10.1016/j.physio.2015.11.005, PMID: 27095482

[37] Nielsen G Stone J Matthews A Brown M Sparkes C Farmer R. Physiotherapy for functional motor disorders: a consensus recommendation. J Neurol Neurosurg Psychiatry. (2014) 86:1113–9. doi: 10.1136/jnnp-2014-309255 25433033 PMC4602268

[38] Badau A Rachita A Sasu CR Clipa A. Motivations and the level of practicing physical activities by physio-kinetotherapy students. Educ. Sci. (2018) 8:97. doi: 10.3390/educsci8030097

[39] Monticone M Ferrante S Rocca B Salvaderi S Fiorentini R Restelli M. Home-based functional exercises aimed at managing kinesiophobia contribute to improving disability and quality of life of patients undergoing total knee arthroplasty: a randomized controlled trial. Arch Phys Med Rehabil. (2013) 94:231–9. doi: 10.1016/j.apmr.2012.10.003, PMID: 23063624

[40] Monticone M Ambrosini E Rocca B Magni S Brivio F Ferrante S. A multidisciplinary rehabilitation programme improves disability, kinesiophobia and walking ability in subjects with chronic low back pain: results of a randomised controlled pilot study. Eur Spine J. (2014) 23:2105–13. doi: 10.1007/s00586-014-3478-5, PMID: 25064093

[41] Hughes CM. Medication non-adherence in the elderly: how big is the problem? Drugs Aging. (2004) 21:793–811. doi: 10.2165/00002512-200421120-00004 15382959

[42] MacNee W Rabinovich RA Choudhury G. Ageing and the border between health and disease. Eur Respir J. (2014) 44:1332–52. doi: 10.1183/09031936.00134014 25323246

[43] Fozard JL Metter EJ Brant LJ Pearson JD Baker GT. Physiology of aging. H Bouma JAM Graafmans Gerontechnol. Stud. Health Technol. Series. (1992) 3:141–67. doi: 10.3233/978-1-60750-847-2-177

[44] Goldspink DF. Ageing and activity: their effects on the functional reserve capacities of the heart and vascular smooth and skeletal muscles. Ergonomics. (2005) 48:1334–51. doi: 10.1080/00140130500101247, PMID: 16338704

[45] Lynch J Smith GD. A life course approach to chronic disease epidemiology. Annu Rev Public Health. (2005) 26:1–35. doi: 10.1146/annurev.publhealth.26.021304.144505 15760279

[46] Shaw BA Krause N Chatters LM Connell CM Ingersoll-Dayton B. Emotional support from parents early in life, aging, and health. Psychol Aging. (2004) 19:4–12. doi: 10.1037/0882-7974.19.1.4 15065927

